# Comprehensive investigation of key biomarkers and pathways in hepatitis B virus-related hepatocellular carcinoma

**DOI:** 10.7150/jca.31287

**Published:** 2019-09-07

**Authors:** Xiwen Liao, Tingdong Yu, Chengkun Yang, Ketuan Huang, Xiangkun Wang, Chuangye Han, Rui Huang, Xiaoguang Liu, Long Yu, Guangzhi Zhu, Hao Su, Wei Qin, Jianlong Deng, Xianmin Zeng, Bowen Han, Quanfa Han, Zhengqian Liu, Xin Zhou, Junqi Liu, Yizhen Gong, Zhengtao Liu, Jianlv Huang, Lei Lu, Xinping Ye, Tao Peng

**Affiliations:** 1 Department of Hepatobiliary Surgery, The First Affiliated Hospital of Guangxi Medical University, Nanning, 530021, Guangxi Zhuang Autonomous Region, People's Republic of China; 2 Department of Hematology, The First Affiliated Hospital of Guangxi Medical University, Nanning, 530021, Guangxi Zhuang Autonomous Region, People's Republic of China; 3 Department of Hepatobiliary Surgery, Affiliated Hospital of Guangdong Medical University, Zhanjiang, 524001, Guangdong Province, China;; 4 Department of Hepatobiliary and Pancreatic Surgery, The First Affiliated Hospital of Zhengzhou University, Zhengzhou, 450000, Henan Province, China;; 5 Department of Hepatobiliary Surgery, The Sixth Affiliated Hospital of Guangxi Medical University, Yulin, 537000, Guangxi Zhuang Autonomous Region, People's Republic of China; 6 Department of Colorectal and Anal Surgery, The First Affiliated Hospital of Guangxi Medical University, Nanning, 530021, Guangxi Zhuang Autonomous Region, People's Republic of China.; 7 Department of Evidence-based Medicine, The First Affiliated Hospital of Guangxi Medical University, Nanning, 530021, Guangxi Zhuang Autonomous Region, People's Republic of China; 8 Key Laboratory of Combined Multi-Organ Transplantation, Ministry of Public Health and Key Laboratory of Organ Transplantation of Zhejiang Province, Hangzhou, 310003, Zhejiang Province, People's Republic of China.; 9 Science for Life Laboratory, KTH-Royal Institute of Technology, Stockholm, SE-171 21, Sweden.; 10 Department of Hepatobiliary Surgery, The Third Affiliated Hospital of Guangxi Medical University, Nanning 530031, Guangxi Zhuang Autonomous Region, People's Republic of China.; 11 Department of General Surgery, Beijing Haidian Hospital, Beijing Haidian Section of Peking University Third Hospital, Beijing, 100080, People's Republic of China.

**Keywords:** hepatitis B virus, hepatocellular carcinoma, DNA topoisomerase II alpha, protein regulator of cytokinesis 1, prognosis

## Abstract

**Objective:** Our study is aim to explore potential key biomarkers and pathways in hepatitis B virus (HBV)-related hepatocellular carcinoma (HCC) using genome-wide expression profile dataset and methods.

**Methods:** Dataset from the GSE14520 is used as the training cohort and The Cancer Genome Atlas dataset as the validation cohort. Differentially expressed genes (DEGs) screening were performed by the *limma* package. Gene set enrichment analysis (GSEA), weighted gene co-expression network analysis (WGCNA), gene ontology, the Kyoto Encyclopedia of Genes and Genomes, and risk score model were used for pathway and genes identification.

**Results:** GSEA revealed that several pathways and biological processes are associated with hepatocarcinogenesis, such as the cell cycle, DNA repair, and p53 pathway. A total of 160 DEGs were identified. The enriched functions and pathways of the DEGs included toxic substance decomposition and metabolism processes, and the P450 and p53 pathways. Eleven of the DEGs were identified as hub DEGs in the WGCNA. In survival analysis of hub DEGs, high expression of *PRC1* and *TOP2A* were significantly associated with poor clinical outcome of HBV-related HCC, and shown a good performance in HBV-related HCC diagnosis. The prognostic signature consisting of *PRC1* and *TOP2A* also doing well in the prediction of HBV-related HCC prognosis. The diagnostic and prognostic values of *PRC1* and *TOP2A* was confirmed in TCGA HCC patients.

**Conclusions:** Key biomarkers and pathways identified in the present study may enhance the comprehend of the molecular mechanisms underlying hepatocarcinogenesis. Additionally, mRNA expression of *PRC1* and *TOP2A* may serve as potential diagnostic and prognostic biomarkers for HBV-related HCC.

## Introduction

Hepatitis B virus (HBV) is prevalent in China and the chronic HBV infection rate in the adult population of this region is between 5% and 10% 1-5. HBV infection is a major cause of hepatocarcinogenesis and increase the risk of death from HBV-related cirrhosis and liver cancer 6-8. According to global cancer statistics, more than half of new liver cancer cases and deaths were from China, in 2012 9. The latest cancer statistics data from China estimated that there will be ~466,100 newly diagnosed liver cancer cases, and ~ 422,100 will die from liver cancer in 2015 10. Liver cancer has become the third major factor of cancer-related death, and with a low 5-year survival rate 10, 11. The histologic type of most liver cancer cases are hepatocellular carcinoma (HCC) 12.

The occurrence of liver cancer is the result of the interaction between genetic factors and environmental factors 6, 7. Genomic analysis is a promising approach to screening potential diagnostic and prognostic biomarkers for diseases, including cancer. Recently, numerous studies use the whole-genome dataset to identify the diagnostic and prognostic molecular markers of HCC, especially the HCC dataset from The Cancer Genome Atlas (TCGA) research network and Gene Expression Omnibus (GEO) database 13-15. The gene expression analyses of human HCCs have led to the successful molecular classification of HCCs on the basis of prognosis, etiology, and intrahepatic recurrence 6. Further comprehensive genomic analysis of the HCC whole-genome dataset to investigate diagnostic and prognostic biomarkers is urgently needed. The aim of our current study is to investigate potential key genes and pathways in HBV-related HCC using bioinformatics approaches base on genome-wide expression profile array, and explore their potential values in HBV-related HCC diagnosis and prognosis.

## Materials and Methods

### Microarray data

GSE14520 dataset and corresponding clinical profiles of HBV-related HCC patients were collected from the GEO database (https://www.ncbi.nlm.nih.gov/geo/query/acc.cgi?acc=GSE14520; accessed August 15, 2017) 14, 15. To avoid a batch effect, the cohort 2 testing group of GSE14520, which was processed on the Affymetrix HT Human Genome U133A Array that consisted of 445 HCC samples, was used for subsequent analysis. Most of these tissues were collected from HBV-related HCC patients. The expression profile array normalization was using *limma* package 16. For multiple probe sets of one gene, the average values of these probe sets are considered as the expression value of this gene 17.

### Gene set enrichment analysis (GSEA)

Differences in pathways and functions between HBV-related HCC tumor and adjacent normal tissues were investigated by GSEA v2-2.2.3 and Molecular Signatures Database (MSigDB) of c2 (c2.cp.kegg.v5.2.symbols.gmt) and c5 (c5.all.v6.0.symbols.gmt), respectively 18-20. Single gene GSEA analysis was also used to explore the molecular mechanisms of different gene expression levels in HCC 20, 21. The parameter of permutations was set at 1,000. The results of significance should meet the criteria of nominal *P*-value<0.05, false discovery rate (FDR)<0.25^20^.

### Identification of differentially expressed genes

Differentially expressed genes (DEGs) between HBV-related HCC tumor and adjacent normal tissues needs to satisfy the following criterions: | log2 fold change (FC) | ≥ 2, *P*-value < 0.05 and FDR < 0.05.

### Functional assessment of DEGs

Gene ontology (GO) and Kyoto Encyclopedia of Genes and Genomes pathway (KEGG) functional assessment of DEGs were used the Database for Annotation, Visualization and Integrated Discovery (DAVID; https://david.ncifcrf.gov/home.jsp; accessed August 15, 2017) v6.8, which is a functional annotation tool for specified genes 22, 23. Enrichment results with *P*-value < 0.05 was considered achieve statistical significance.

### Weighted gene co-expression network analysis (WGCNA)

Co-expression analysis of DEGs and hub DEGs identification were performed by WGCNA 24. Gene-gene correlation coefficients of WGCNA greater than 0.2 were incorporated into subsequent hub DEG identification and weighted co-expression network mapping. Node degrees represent the power of the connection between the selected node and others in the network are used for hub DEG identification.

### Prognostic signature Investigation and validation

We further investigated the potential role of these hub DEGs in HBV-related HCC survival and recurrence. Each hub DEG was assessed by a multivariate Cox proportional hazards regression model that was adjusted for age, gender, cirrhosis, BCLC stage, and serum α-fetoprotein (AFP) level in the GSE14520 cohort. The hub DEGs that correlated with the HBV-related HCC prognosis were applied to constructed prognostic signature and was established on the basis of a prognosis risk score: gene expression value multiplied by a regression coefficient (β), which was generated from a multivariate Cox proportional hazards regression model. The risk score is calculated as below 25-28: risk score = expression of Gene_1_ × β_1_Gene_1_ + expression of Gene_2_ × β_2_Gene_2_ +… expression of Gene_n_ × β_n_Gene_n_. The time-dependent receiver operating characteristic (ROC) curve was used the *survivalROC* package (https://CRAN.R-project.org/package=survivalROC) to assess the accuracy of prognostic signature 29. For validation and to generalize this prognostic signature, HCC patients from TCGA (https://portal.gdc.cancer.gov/; accessed August 15, 2017) were regarded as an independent validation cohort.

### Statistical analysis

The test used to compare the expression between two groups was assessed by independent sample t-test. The FDR in DEG screening and GSEA were performed according to the Benjamini-Hochberg procedure 30-32. Volcano plots, as well as heat maps were constructed with the *gplots* package. The weighted co-expression networks were drawn by Cytoscape version 3.4.0 (http://www.cytoscape.org/; accessed January 16, 2017) 33. Univariate survival analysis was assessed by the Kaplan-Meier method with the log-rank test. Hazard ratio (HR) and 95% confidence interval (CI) were derived from the multivariate Cox proportional hazards regression model after data in the GSE14520 was adjusted for age, gender, cirrhosis, BCLC stage, and serum AFP levels, whereas the TCGA cohort data were adjusted for age, gender, and tumor stage. ROC curves were used to assess the sensitivity of DEGs in distinguishing HCC tumor tissue from adjacent normal tissue. *P*-value < 0.05 was considered achieve statistical significance. SPSS version 20.0 (IBM Corporation, Armonk, NY, USA) and R 3.3.0 were performed the statistical analyses.

## Results

### Study population

When screening the 445 samples of the GSE14520 Affymetrix HT Human Genome U133A Array dataset, the inclusion criteria were as follows: (i) patients with HBV infection, (ii) patients with complete prognostic information. We excluded the samples from patients without HBV infection or patients with an unavailable HBV infection record in the GSE14520 supplementary material, as well as patients with unavailable prognostic parameters. A total of 212 tumor samples and 204 adjacent normal tissue samples were included for further study. Clinical features of HBV-related HCC patients from the GSE14520 cohort are shown in **Table 1.**

### GSEA between HCC tumor and adjacent normal tissues

**Figure 1:**
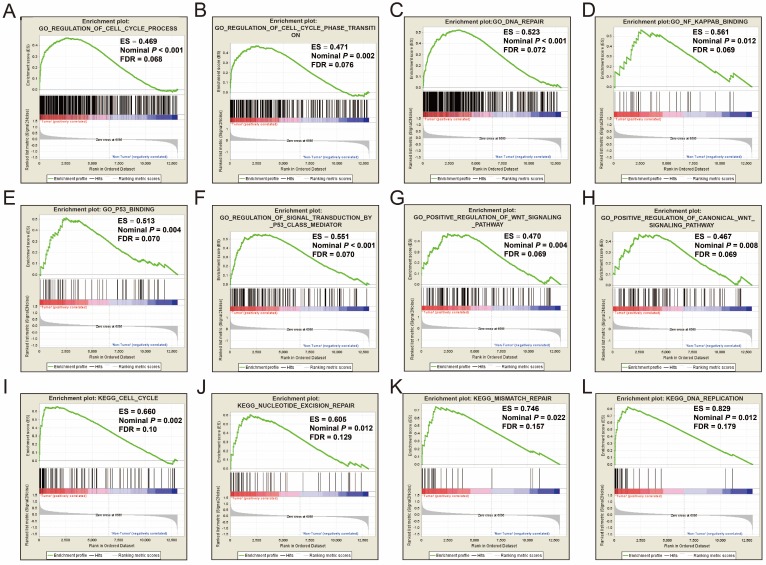
GSEA results of tumor and adjacent normal tissue in HBV-related HCC patients of GSE14520 cohort. GSEA result of HBV-related HCC tumor tissue using c2 (A-H) and c5 (I-L) reference gene sets.

GSEA was performed on the c5 reference gene set (GO gene set), the GO enrichment results demonstrated that cell cycle, DNA repair, nuclear factor-kappa B (NF-κ B), p53, and Wnt signaling pathway were significantly enriched in tumor tissue (**Figure 1A-H**). Consistent with the c5 reference gene set analysis, the c2 reference gene set (KEGG gene set) GSEA suggests that genes involved in the cell cycle, nucleotide excision repair, mismatch repair and DNA replication pathways were significantly enriched in tumor tissues (**Figure 1I-L**). All the significant GSEA results from the c2 and c5 reference gene set can be found in **Tables S1 and S2**, respectively.

### Identification of DEGs

**Figure 2:**
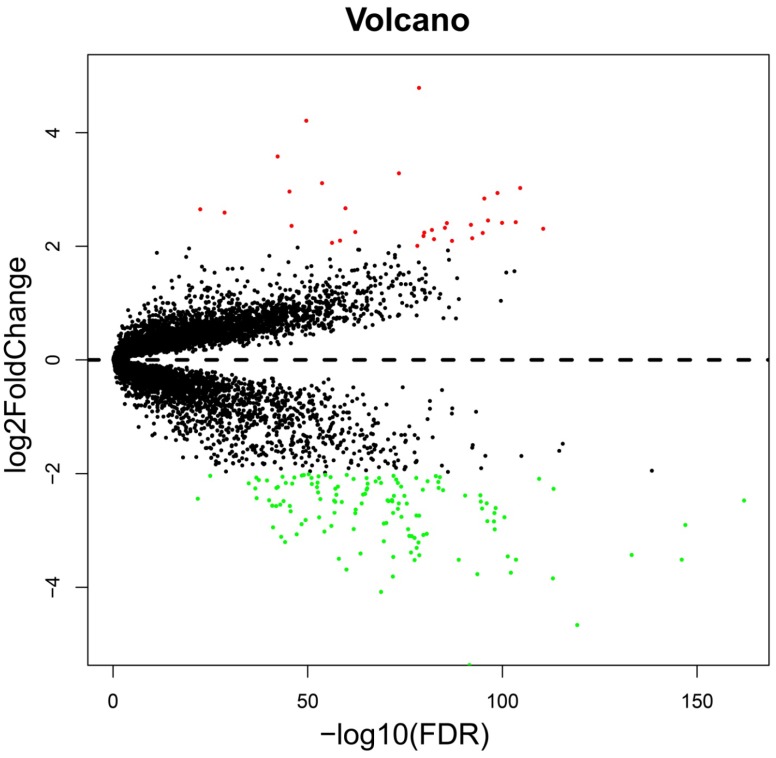
Volcano plot of the DEGs. Red dots: up-regulation; green dots: down-regulation; black dots: non-differentially expressed genes.

**Figure 3:**
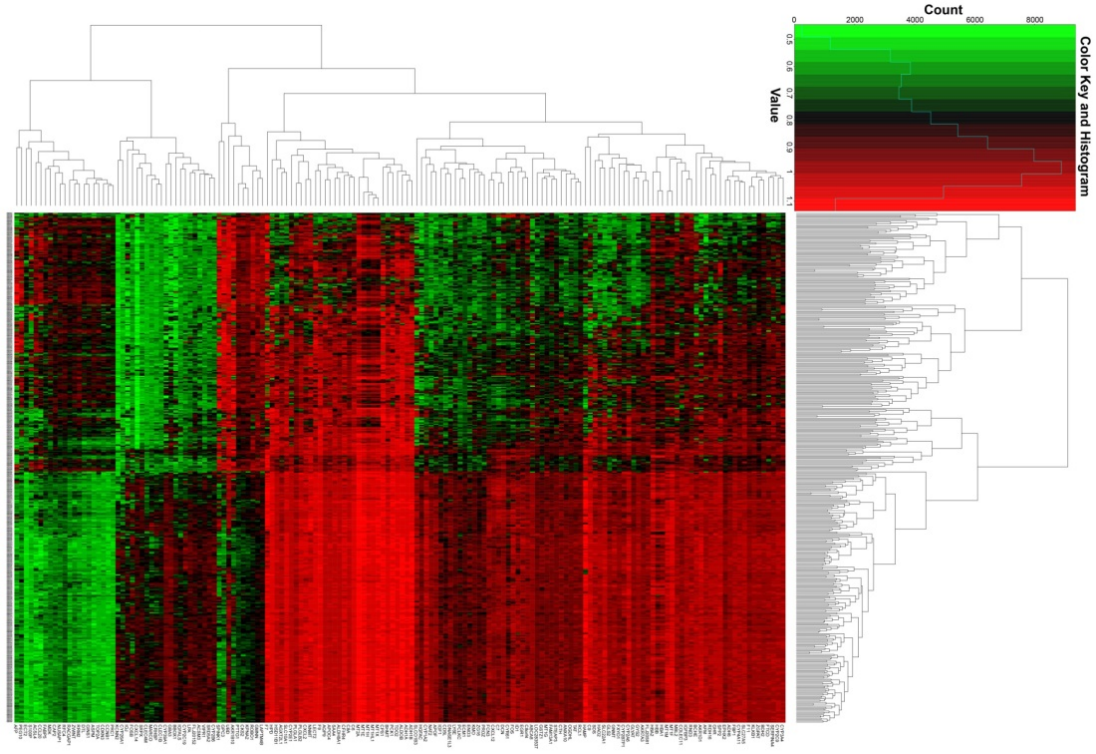
Heat map of the 160 DEGs.

The whole-genome expression profile chip dataset of the 212 tumor and 204 adjacent normal tissue samples were compared in the R platform by using the *limma* package. There were 160 genes that met the DEG criterion (**Table S3**), of which 31 were upregulated and 129 were downregulated. Among these DEGs, a well-known biomarker for HCC diagnosis and prognosis, AFP, was identified (log2FC = 2.65 and FDR = 4.87×10^-23^,** Table S3**). A volcano plot of the DEGs is shown in **Figure 2**, and a heat map of the DEGs is shown in **Figure 3**.

### Functional assessment of DEGs

**Figure 4:**
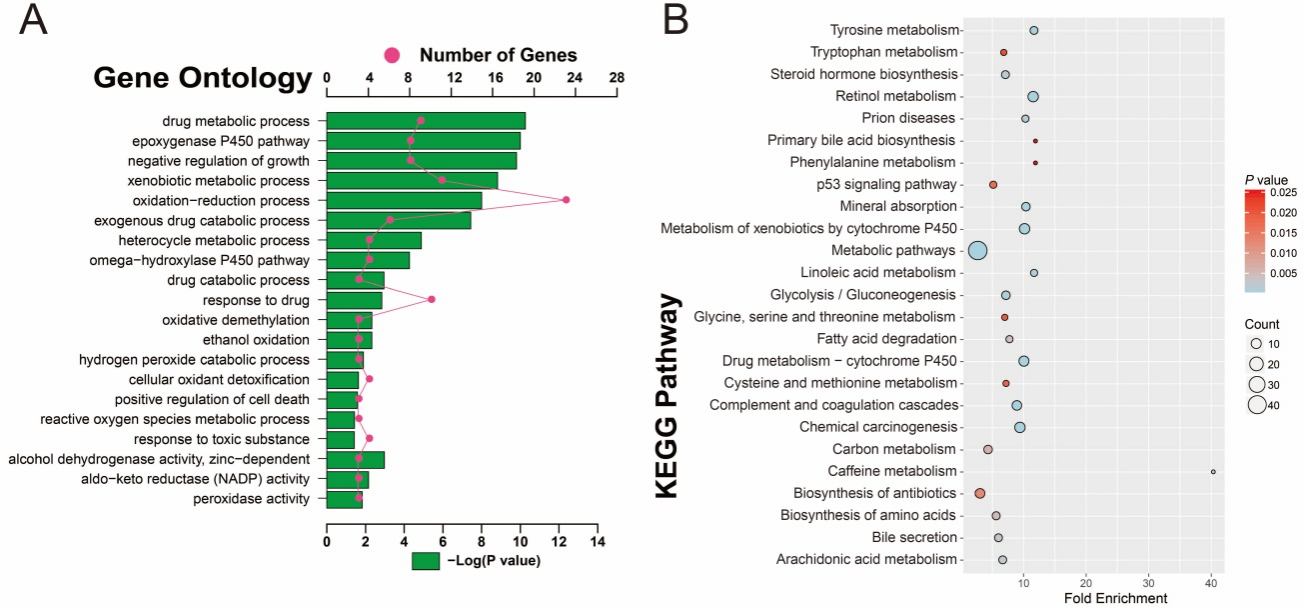
GO and KEGG enrichment results of 160 DEGs. (A) GO term enrichment results. (B) KEGG enrichment results.

To investigate the potential function and pathways that are associated with HBV-related HCC tumorigenesis, 160 DEGs were analyzed with DAVID. GO term analysis (**Table S4 and Figure 4A**) indicated that these DEGs were mainly involved in toxic substance decomposition and metabolism-related biological processes, such as drug metabolic and catabolic processes, the P450 pathway, ethanol oxidation, hydrogen peroxide catabolic process, oxidative demethylation, cellular oxidant detoxification, peroxidase and alcohol dehydrogenase activity, oxidation-reduction process, and response to toxic substances, in addition to regulation of cell death and growth. Consistent with the GO term enrichment results, KEGG enrichment results (**Table S5 and Figure 4B**) also suggest that the DEGs were mainly involved in substance decomposition and metabolism-related biological processes, such as retinol metabolism, drug metabolism, the cytochrome P450 pathway, carbon metabolism, arachidonic acid metabolism, cysteine and methionine metabolism, glycine, serine and threonine metabolism, tryptophan metabolism and phenylalanine metabolism. The p53 signaling pathway, which is associated with the regulation of cell death, was also enriched, as well as the metabolism-related carcinogenic pathway and chemical carcinogenesis.

### WGCNA and hub DEGs screening

**Figure 5:**
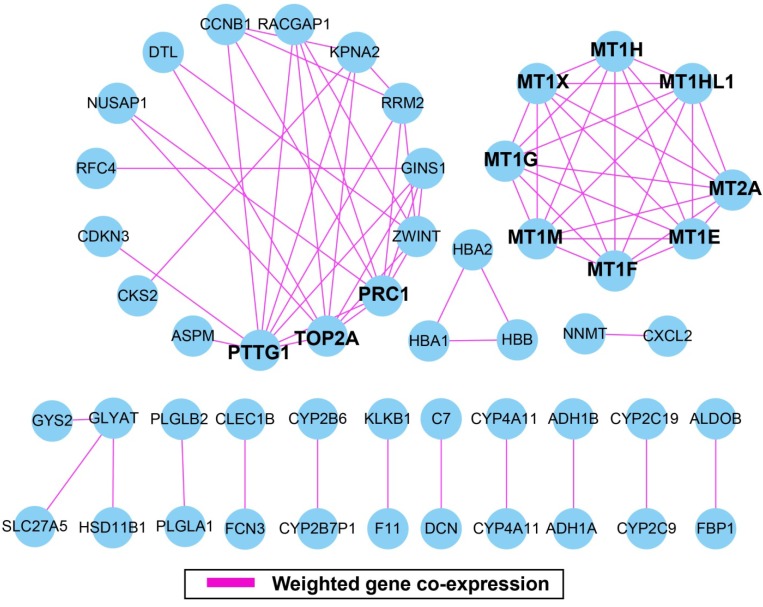
Weighted gene co-expression network. Blue circles represent differentially expressed genes.

The weighted gene co-expression correlation coefficient between two genes greater than 0.2 was used for weighted gene co-expression network construction. A total of 50 DEGs and 75 edges were exported for final network construction (**Figure 5, Table S6**). The top-ten degree DEGs of the weighted gene co-expression network were identified as hub DEGs. These hub DEGs included pituitary tumor-transforming 1 (*PTTG1*), DNA topoisomerase II alpha (*TOP2A*), protein regulator of cytokinesis 1 (*PRC1*), metallothionein 2A (*MT2A*), metallothionein 1X (*MT1X*), metallothionein 1M (*MT1M*), metallothionein 1H (*MT1H*), metallothionein 1G (*MT1G*), metallothionein 1F (*MT1F*), metallothionein 1E (*MT1E*), and metallothionein 1H like 1 (*MT1HL1*). Among these hub genes, *PTTG1* showed the highest node degree, which was 9 (**Figure 5**).

### Construction of a prognostic signature in the GSE14520 cohort

To explore the values of these hub DEGs in HBV-related HCC clinical outcome, we also used the multivariate survival analysis to identify prognostic hub DEGs. Low- and high-expression of a selected gene were group by the median value of this gene. We subsequently used the multivariate Cox proportional hazards regression model analysis in the GSE14520 cohort by adjusting for age, gender, cirrhosis, BCLC stage and serum AFP levels. *PRC1*and *TOP2A* were identified as the prognostic hub DEGs and used for further investigation.

**Figure 6:**
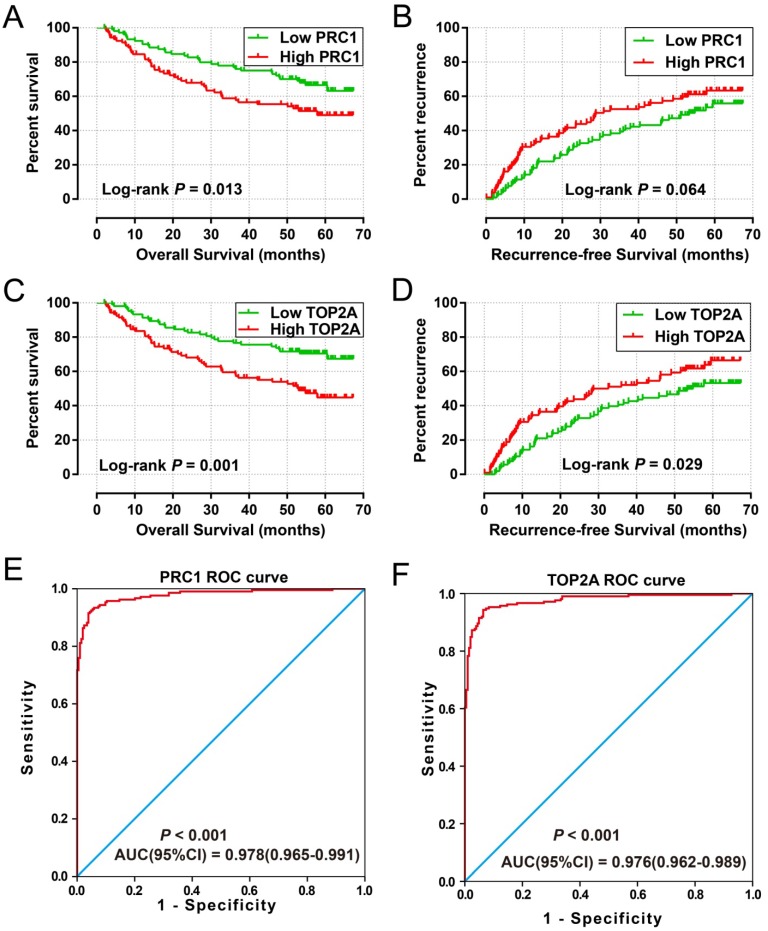
Prognostic and diagnosis value of *PRC1* and *TOP2A* in GSE14520 cohort. Kaplan-Meier curve of *PRC1* expression in OS (A) and RFS (B); Kaplan-Meier curve of *TOP2A* expression in OS (C) and RFS (D); ROC curves of the *PRC1* (E) and *TOP2A* (F) to distinguish HBV-related HCC tissue from adjacent normal tissue.

High expression of *PRC1* (adjusted P = 0.039, adjusted HR = 1.490, 95% CI = 1.020-2.176 for recurrence-free survival [RFS]; adjusted P = 0.007, adjusted HR = 1.862, 95% CI = 1.188-2.919 for overall survival [OS];** Table 2 and Table 3, Figure 6A-B**) and* TOP2A* (adjusted P = 0.045, adjusted HR = 1.472, 95% CI = 1.009-2.146 for RFS; adjusted P = 0.002, adjusted HR = 2.027, 95% CI = 1.284-3.201 for OS; **Table 2 and Table 3, Figure 6C-D**) was significantly associated with an increased risk of death and recurrence. ROC curve analysis indicates that* PRC1* (*P* < 0.001, area under curve [AUC] = 0.976, 95%CI = 0.962-0.989) and* TOP2A* (*P* < 0.001, AUC = 0.978, 95%CI = 0.965-0.991) performed well in discriminate the HBV-related HCC tumor and adjacent normal tissue.

To investigate the role of *PRC1*and *TOP2A* in HCC, the expression distribution of *PRC1* and *TOP2A* in human normal tissue were analyzed using the GTEx Portal website (https://www.gtexportal.org/home/; accessed August 15, 2017) 34. Both the *PRC1* and *TOP2A* expression level in human normal liver tissue was low compared with other human organs (**Figure S1A, B**). Furthermore, we also investigate the expression of *PRC1*and *TOP2A* between normal liver and HCC tumor tissues by using the Human Protein Atlas website (https://www.proteinatlas.org/; accessed February 2, 2018) 35, 36, and found that both the protein expression of *PRC1*and *TOP2A* were upregulated in HCC tumor tissue, which detected by immunohistochemistry (**Figure S1C-F**). Whereas, the expression of *PRC1* and *TOP2A* in HBV-related HCC tumor tissue was markedly upregulated (**Figure S2A**) in the GSE14520 cohort, and also up-regulation in advance tumor stage samples (**Figure S2B**).

Genes that have different expression between normal liver and HCC tumor tissue could potentially be used in HCC diagnosis. ROC analysis suggested that *PRC1* and *TOP2A* performed well in HCC diagnosis. In addition, the co-expression analysis performed by Pearson correlation also suggest that expression of *PRC1* and *TOP2A* had a strong correlation (r = 0.798, *P* < 0.0001) in both HBV-related HCC tumor tissue and adjacent normal tissue (r =0.565, *P* <0.0001). Therefore, the combination of *PRC1* and *TOP2A* in HBV-related HCC prognosis are worth further study.

**Figure 7:**
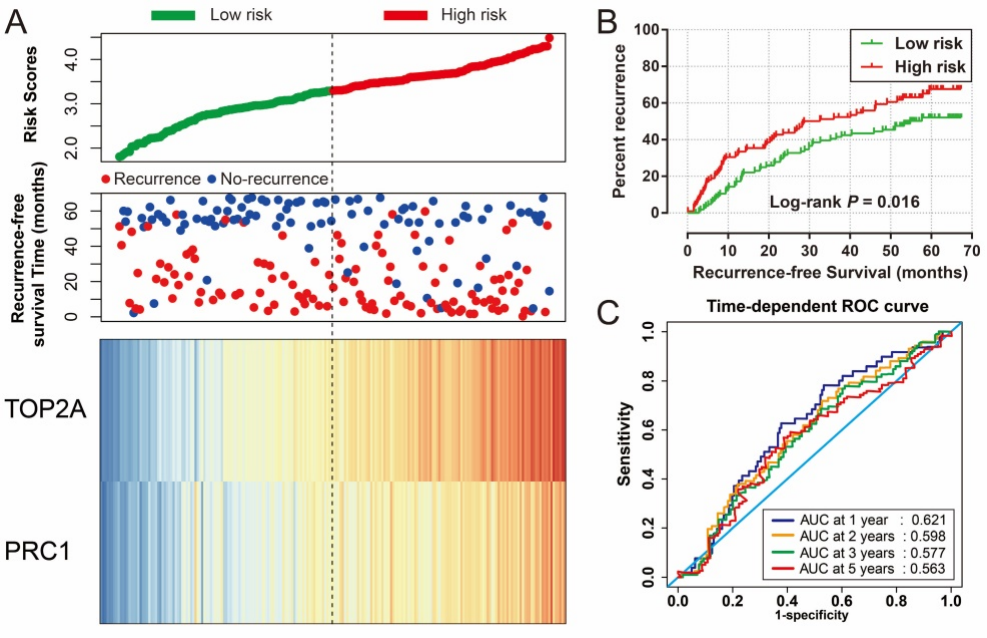
Prognostic risk score model analysis of *PRC1* and *TOP2A* signature in RFS of GSE14520 cohort. (A) Scatter plots of risk score and survival time, and *PRC1* and *TOP2A* gene expression heat map. (B) Kaplan-Meier curves for low- and high-risk score. (C) Time-dependent ROC curve of risk score.

**Figure 8:**
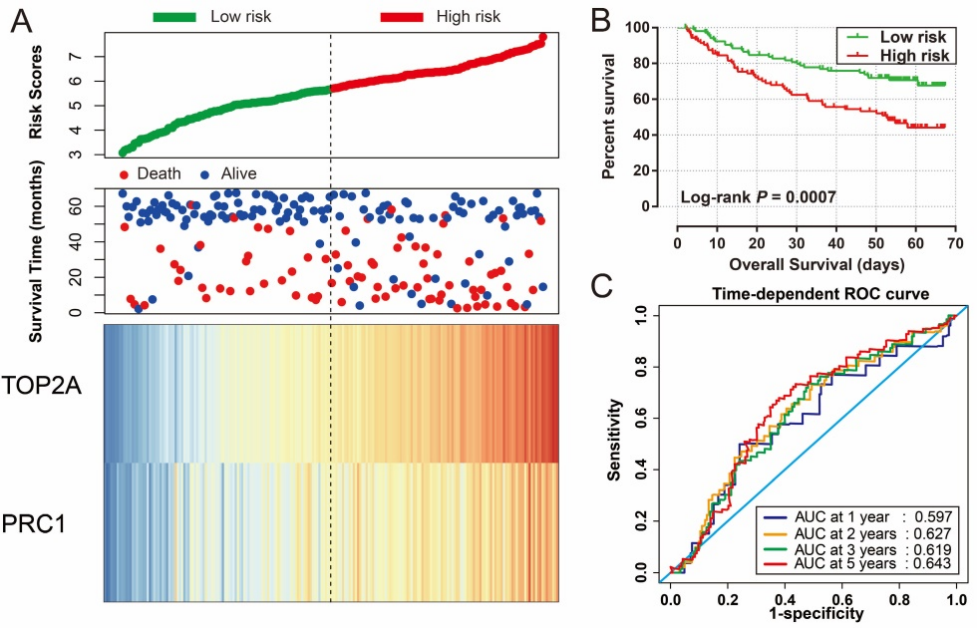
Prognostic risk score model analysis of *PRC1* and *TOP2A* signature in OS of GSE14520 cohort. (A) Scatter plots of risk score and survival time, and *PRC1* and *TOP2A* gene expression heat map. (B) Kaplan-Meier curves for low- and high-risk score. (C) Time-dependent ROC curve of risk score.

Risk score of HBV-related HCC prognostic signature was consisted of PRC1 and TOP2A expression and weighted by the regression coefficient (β). The formulas of the risk score for RFS and OS were as follows: risk score (RFS) = 0.311 × expression of *TOP2A* + 0.189 × expression of *PRC1*; risk score (OS) = 0.628×expression of* TOP2A* + 0.231×expression of *PRC1*. Low- and high-risk groups were group by the median value of the risk score. Survival analysis revealed that patients with a high risk score had a higher recurrence (adjusted *P* = 0.029, adjusted HR = 1.525, 95% CI = 1.045-2.224; **Table 4, Figure 7A, B**) and death (adjusted* P* = 0.002, adjusted HR = 2.029, 95% CI = 1.287-3.198; **Table 4, Figure 8A, B**) risk. Time-dependent ROC analysis revealed that the risk score doing well in HBV-related HCC prognosis, with the AUC of ROC curves were 0.621, 0.598, 0.577 and 0.563 for 1-, 2-, 3- and 5-year recurrence (**Figure 7C**), respectively. For 1-, 2-, 3- and 5-year survival, the AUC of ROC curves were 0.597, 0.627, 0.619 and 0.643 (**Figure 8C**), respectively.

### Validation of the prognostic signature in TCGA cohort

**Figure 9:**
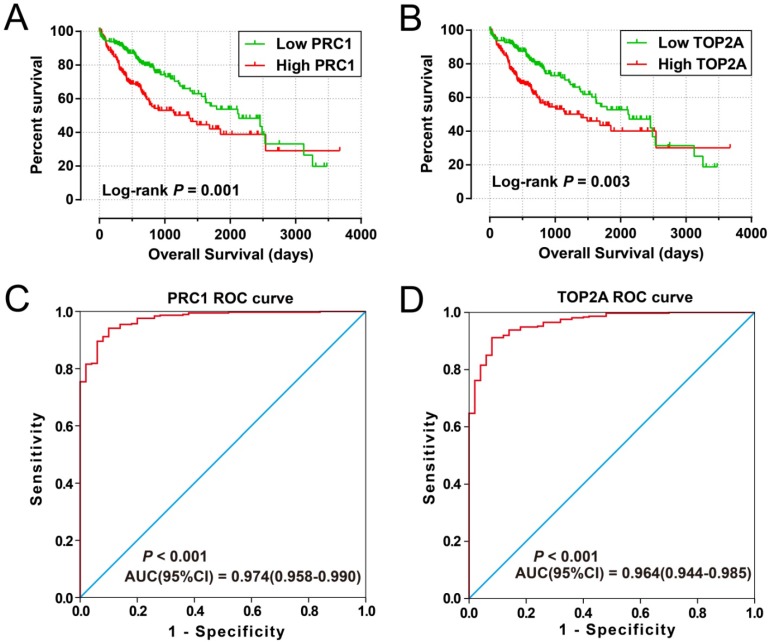
Prognostic and diagnosis value of *PRC1* and *TOP2A* in TCGA cohort. Kaplan-Meier curve of *PRC1* expression in OS (A); Kaplan-Meier curve of *TOP2A* expression in OS (B); ROC curves of the *PRC1* (C) and *TOP2A* (D) to distinguish HCC tissue from adjacent normal tissue;

**Figure 10:**
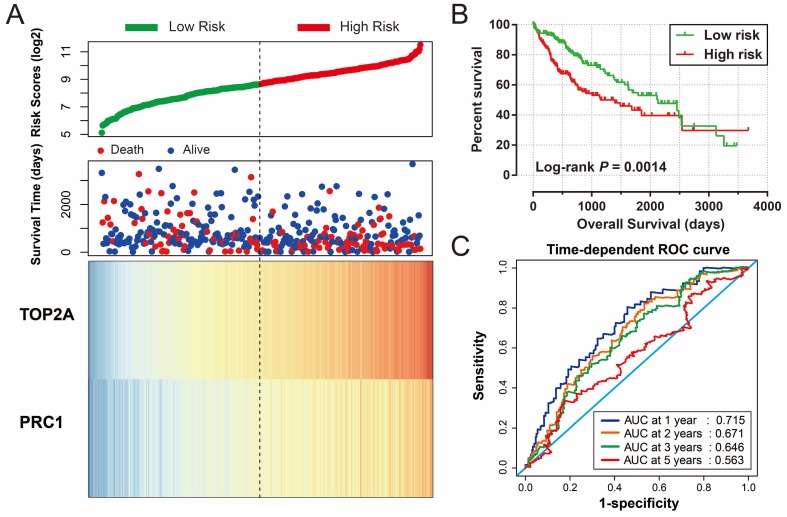
Prognostic risk score model analysis of *PRC1* and *TOP2A* signature in OS of TCGA cohort. (A) Scatter plots of risk score and survival time, and *PRC1* and *TOP2A* gene expression heat map. (B) Kaplan-Meier curves for low- and high-risk score. (C) Time-dependent ROC curve of risk score.

For validation and to generalize the prognostic signature, HCC patients from TCGA were considered as the validation cohort. These patients from the TCGA who received RNA sequencing and with a complete prognostic parameters were included into further survival analysis. The expression of *PRC1* and *TOP2A* also showed a strong correlation in HCC samples from TCGA (r = 0.922, *P* < 0.0001 for HCC tumor tissues, and r =0.842, *P* <0.0001 for adjacent normal tissues). Because the clinical features from the TCGA cohort was incomplete, only data concerning the parameters of age, gender, tumor stage, and OS time and status were available from the TCGA website (**Table 5**). Thus, only the prognostic signature of the OS could be validated in the current study. Survival analysis revealed that both the high expression of *PRC1* (adjusted *P* = 0.002, adjusted HR = 1.817, 95% CI = 1.235-2.673; **Table 6, Figure 9A**) and *TOP2A* (adjusted *P* = 0.015, adjusted HR = 1.619, 95% CI =1.097-2.390; **Table 6, Figure 9B**) were markedly raise the risk of death, consistent with the results of GSE14520. Similar to the validation of survival analysis, ROC analysis of *PRC1* (*P* < 0.001, AUC = 0.974, 95% CI = 0.958-0.990, **Figure 9C**) and *TOP2A* (*P* < 0.001, AUC = 0.964, 95% CI = 0.944-0.985, **Figure 9D**) in TCGA also performed well in distinguishing the HCC tumor and adjacent normal tissues. Comparison of gene expression in TCGA cohort HCC samples also showed that *PRC1* and *TOP2A* were significant up-regulation in HCC tumor tissues (**Figure S2C**) and up-regulation in advance tumor stage tumor tissues (**Figure S2D**). Survival analysis suggest that patients with high risk score were markedly associated with a higher risk of death (adjusted *P* = 0.011, adjusted HR = 1.648, 95% CI = 1.120-2.427; **Table 6, Figure 10A, B**), while survivalROC analysis of risk score also doing well in HCC OS prediction, as the AUC of ROC curves were 0.715, 0.671, 0.646 and 0.563 for 1-, 2-, 3- and 5-year survival (**Figure 10C**), respectively.

### Single gene GSEA of *PRC1* and *TOP2A*

In order to explored the potential molecular mechanisms of different *PRC1* and *TOP2A* gene expression groups in HCC prognosis, therefore, single gene GSEA analysis was used to investigated the potential pathways and biological processes between different gene expression groups in both GSE14520 and TCGA HCC cohorts.

**Figure 11:**
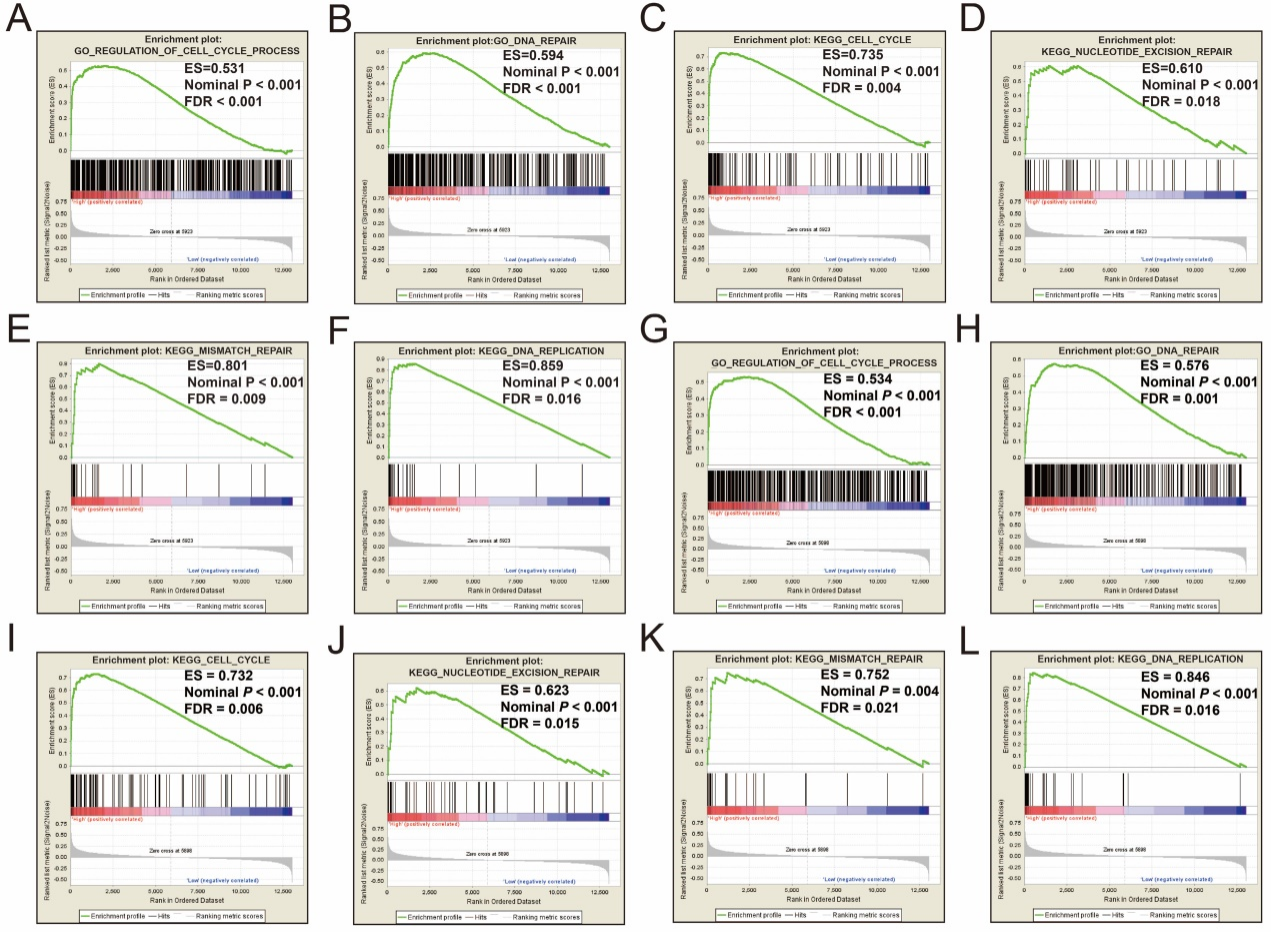
GSEA results between different *PRC1* and *TOP2A* gene expression levels in GSE14520 cohort. GSEA results of high *PRC1* expression groups in GSE14520 cohort (A-F); GSEA results of high *TOP2A* expression groups in GSE14520 cohort (G-L).

**Figure 12:**
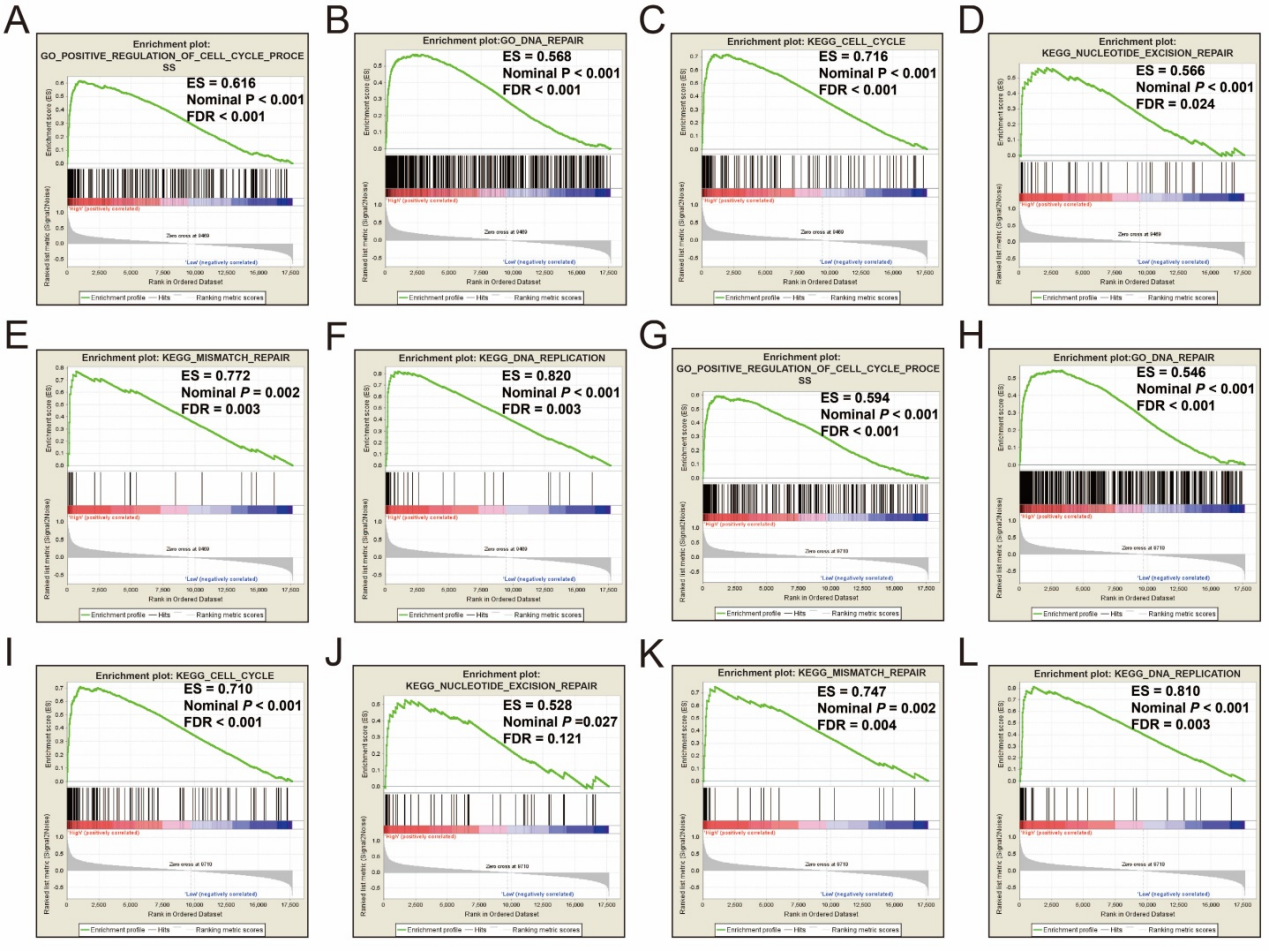
GSEA results between different *PRC1* and *TOP2A* gene expression levels in HCC patients of TCGA cohort. GSEA results of high *PRC1* expression groups in HCC patients of TCGA cohort (A-F); GSEA results of high *TOP2A* expression groups in HCC patients of TCGA cohort (G-L).

Single gene GSEA analysis suggest that both the high expression of *PRC1* and *TOP2A* were markedly enriched in the cell cycle and DNA repair biological processes in GSE14520 (**Figure 11A-L, Table S7-10**) and TCGA (**Figure 12A-L, Table S11-14**) cohorts, which consistent with the GSEA analysis results between HBV-related HCC tumor and adjacent normal tissues in GSE14520.

## Discussion

The liver is an organ that metabolites toxic substances. Dysregulation of the genes related to toxic material metabolism will lead to hepatocarcinogenesis. Investigation of hepatocarcinogenesis by GSEA suggest that the cell cycle 37, DNA repair 38, NF-κ B signaling pathway 39, p53 pathway 40 and Wnt signaling pathway 41 were associated with hepatocarcinogenesis, which has already been reported in previous studies. In addition, GO and KEGG enrichment analysis demonstrated that the biological processes of toxic substance decomposition and metabolism processes (including drugs and ethanol), P450 pathway 42, 43, p53 pathway 40, alcohol dehydrogenase activity and cell death regulation in the functional assessment of DEGs have also been reported in previous studies in connection with HCC. These DEGs associated with the organ function of the liver may play a crucial part in the development of hepatocarcinogenesis.

Most of the 11 hub DEGs of the WGCNA network have been reported to be related to HCC in previous studies. Work by Tao et al. indicates that the down-regulation of expression in the metallothionein family in HCC may participate in hepatocarcinogenesis and serve as a biomarker for hepatocellular differentiation 44. Previous studies have substantiated that *PTTG1* 45 and *MT1M* 46 were associated with HCC tumorigenesis, furthermore, *MT1H* 47*, MT1M* 48 and* MT1G* 49 serve as tumor suppressor genes in HCC. In addition, studies also suggest that* MT1G* promotes sorafenib resistance and is a biomarker for exploring the impact of sorafenib on the redox metabolism of cancer cells 50, 51.

Numerous studies have shown that *PRC1* and *TOP2A* play roles in multiple cancers and extensive studies have shown that *PRC1* is involved in cell cycle processes. Studies by Mollinari et al. and Zhu et al. have substantiated that *PRC1* is a bundling protein that is essential in maintaining the mitotic spindle midzone and plays a crucial role in midzone formation and cytokinesis 52, 53. Recent research suggests that *PRC1* is down-regulated by p53 in breast cancer cells, genetic variation of *PRC1* is associated with breast cancer susceptibility, and* PRC1* overexpression predicts poor disease-free survival of patients with breast cancer 54-56. Consistent with this breast cancer research, previous studies have also indicated that *PRC1* was up-regulated in tumor tissue and overexpression in tumor tissue promotes early recurrence in patients with HCC and prostate cancer 57, 58. Consistent with the previous studies, our current study suggests that PRC1 was up-regulated in HBV-related HCC tumor tissue, and high *PRC1* expression promotes a poor OS and RFS. In addition, we also explored the diagnostic value of *PRC1* in HBV-related HCC and found that *PRC1* is a promising diagnostic biomarker for HCC.

Several studies indicate that altered expression of *TOP2A* in tumor tissue was a prognostic biomarker for multiple cancers. Previous studies have already substantiated that the high expression of *TOP2A* was markedly related to poor OS and promotes early recurrence in patients with prostate cancer 59, endometrial cancer 60, non-muscle-invasive bladder cancer 61, and adrenocortical carcinoma 62. In addition, high* TOP2A* expression has also been reported in patients with advanced gallbladder carcinoma 63, clear cell renal cell carcinoma 64 and HCC 65 with a significantly increased risk of death. Consistent with the previous studies, our current study also demonstrated that high *TOP2A* expression significantly increased risk of death and recurrence in patients with HCC. In terms of diagnosis, previous studies also reveal that *TOP2A* has notably increased expression in HCC tumor tissues 65, 66 compared to the adjacent normal liver tissues. Our current study further validated this finding; moreover, ROC analysis also indicated that *TOP2A* performed well in HCC diagnosis.

There were numerous previous studies have identified or summarized the key biomarkers for HBV-related HCC, several of them were also have been identified as DEGs between tumor and adjacent normal tissues, such as AFP, glypican 3 (GPC3), midkine (MDK), karyopherin subunit alpha 2 (KPNA2), cyclin B1 (CCNB1) 67-71. The advantages and differences between our current study and previously published papers were that the present study was investigated the hepatocarcinogenesis using the GSEA, and the potential mechanism of *PRC1* and *TOP2A* also revealed by the GSEA. The present study was the first investigation into the hepatocarcinogenesis potential mechanism using GSEA. Multiple previous studies also have identified hub DEGs between HCC tumor and non-tumor tissues, however, the hub DEGs identified by them were performed by the Search Tool for the Retrieval of Interacting Genes/Proteins (STRING) online tool, and the database of STRING cannot represent the tissue specificity of HCC 72-74. So, the hub genes identified by STRING were not real in HCC. The advantages of the hub DEGs in the present study were identified by WGCNA method based on the GSE14520 dataset, and more suitable to exploring the co-expression relationship in HCC tumor tissues then STRING, as well as the results obtained by WGCNA were more reliable then STRING. In addition, due to the strong correlation of *PRC1* and *TOP2A*, we combine the* PRC1* and *TOP2A* through a linear combination method, and then constructed a prognostic signature for HCC prognosis prediction. The prognostic signature of *PRC1* and *TOP2A* combined, doing well in HCC clinical outcome prediction in the GSE14520 cohort and was validated in the cohort from TCGA. ROC analysis of the prognostic signature revealed that the five-year survival rate of HCC OS can be predicted based on the expression of *PRC1* and *TOP2A* through this linear combination method.

The genome-scale dataset provides a promising source for discovering diagnostic and prognostic biomarkers. However, there are still some limitations need statements. First, the clinical features available from the GSE14520 and TCGA database were incomplete. Thus, We evaluate the association between genes and HCC prognosis based on multivariate Cox proportional hazards regression model is only adjusted for age, gender, cirrhosis, BCLC stage, and serum AFP levels in the GSE14520 cohort, whereas, adjusted for age, gender, and tumor stage in TCGA cohort. Second, due to the lack of RFS information in the cohort from TCGA, the present study failed to verify the prognostic signature in HBV-related HCC RFS. And because of the HBV infection status in TCGA are unavailable on the TCGA website, therefore, we failed to identify the DEGs of HBV-related HCC using TCGA HCC cohort. Independent validation cohorts of HCC patients are necessary to evaluate this prognostic signature.

In spite of the limitations, the present study has identified 160 DEGs between HBV-related HCC tumor and adjacent normal tissue, which also include AFP. Bioinformatics analysis by GSEA, GO, KEGG, and the WGCNA network provides an insight into hepatocarcinogenesis and will thus help to develop effective diagnostic and therapeutic strategies. By a comprehensive investigation, we found that both *PRC1* and *TOP2A* may be underlying diagnostic and prognostic biomarkers for HBV-related HCC. Furthermore, the prognostic signature of *PRC1* and *TOP2A* doing well in prognosis prediction of HBV-related HCC. Single gene GSEA analyses indicate that both the high expression of *PRC1* and *TOP2A* were obviously enriched in cell cycle and DNA repair related pathways and biological processes.

## Conclusions

The present study aimed to identify DEGs that may be involved in hepatocarcinogenesis using a genome-scale and bioinformatic analysis to investigate the potential pathways and biological processes involved in HBV-related HCC. A total of 160 DEGs and 11 hub DEGs were identified and may be regarded as diagnostic biomarkers for HBV-related HCC. Several pathways and biological processes, such as toxic substances decomposition and metabolism processes, the cell cycle, the P450 pathway and p53 pathway, may play a critical role in hepatocarcinogenesis and are worthy of further study. The mRNA expression of hub DEGs, *PRC1* and *TOP2A*, were regarded as potential diagnostic and prognostic biomarkers for HBV-related HCC. However, further studies are needed to elucidate the biological function of these genes and pathways in HBV-related HCC, and external validation cohorts are necessary to confirm our findings.

## Supplementary Material

Supplementary figure and table legends, figures.

Supplementary tables.

## Figures and Tables

**Table 1:** ^a^Clinical features of HBV-related HCC patients in GSE14520 cohort

Variables	Patients (n=212)	RFS					OS			
		No. of events	MRT(months)	HR (95% CI)	*P*		No. of events	MST(months)	HR (95% CI)	*P*
**Age**										
≤60	175	96	45	1			69	NA	1	
>60	37	20	48	0.974(0.602-1.578)	0.916		13	NA	0.864(0.478-1.564)	0.63
**Gender**										
Female	29	10	NA	1			8	NA	1	
Male	183	106	40	2.143(1.120-4.100)	0.021		74	NA	1.704(0.821-3.534)	0.152
**Tumor number**										
Single	167	90	49	1			59	NA	1	
Multiple	45	26	28	1.216(0.785-1.883)	0.382		23	47	1.607(0.992-2.604)	0.054
**Tumor Size†**										
≤5cm	137	73	51	1			46	NA	1	
>5cm	74	43	28	1.409(0.966-2.056)	0.075		36	53	1.975(1.274-3.060)	0.002
**Cirrhosis**										
No	17	5	NA	1			2	NA	1	
Yes	195	111	37	2.612(1.066-6.402)	0.036		80	NA	4.335(1.065-17.638)	0.041
**BCLC stage**										
0	20	6	NA	1			2	NA	1	
A	143	74	51	2.050(2.892-4.711)	0.091		48	NA	4.119(1.001-16.951)	0.05
B	22	15	26	4.019(1.550-10.421)	0.004		12	46	8.992(2.005-40.320)	0.004
C	27	21	8	6.163(2.477-15.333)	<0.001		20	13	18.993(4.419-81.632)	<0.001
**Tumor Stage**										
I	89	35	NA	1			20	NA	1	
II	76	48	28	1.995(1.289-3.088)	0.02		32	NA	2.214(1.265-3.873)	0.05
III	47	33	18	3.220(1.993-5.204)	<0.001		30	18	5.197(2.930-9.218)	<0.001
**AFP (ng/mL) ‡**										
≤300	115	62	48	1			39	NA	1	
>300	94	54	35	1.200(0.833-1.728)	0.328		43	NA	1.546(1.002-2.385)	0.049

**Notes:** † Information of tumor size was unavailable in 1 patients; ‡ Information of AFP was unavailable in 3 patients; ^a^ The data in this table also have been shown in our previous publication (Reference 28). RFS, recurrence-free survival; OS, overall survival; MRT, median recurrence time; MST, median survival time; HR, hazard ratio; CI, confidence interval. BCLC, Barcelona Clinic Liver Cancer; AFP, α-fetoprotein.

**Table 2:** Correlation between RFS and 11 hub DEGs expression in HBV-related HCC patients of GSE14520 cohort

Variables	Patients (n=212)	MRT (months)	No. of events	Crude HR (95% CI)	Crude P	Adjusted HR (95% CI)	Adjusted P§
***PTTG1***							
Low	106	51	56	1		1	
High	106	36	60	0.174(1.288(0.895-1.854)	0.174	1.406(0.961-2.057)	0.079
**PRC1**							
Low	106	53	55	1		1	
High	106	28	61	1.409(0.978-2.030)	0.066	1.490(1.020-2.176)	0.039
**TOP2A**							
Low	106	54	53	1		1	
High	106	32	63	1.498(1.039-2.160)	0.03	1.472(1.009-2.146)	0.045
***MT2A***							
Low	106	46	58	1		1	
High	106	43	58	0.949(0.659-1.365)	0.777	0.857(0.591-1.244)	0.418
***MT1X***							
Low	106	40	60	1		1	
High	106	48	56	0.856(0.594-1.232)	0.402	0.785(0.540-1.142)	0.206
***MT1M***							
Low	106	40	60	1		1	
High	106	48	56	0.849(0.589-1.222)	0.377	0.779(0.538-1.129)	0.188
***MT1H***							
Low	106	40	60	1		1	
High	106	48	56	0.854(0.593-1.230)	0.397	0.787(0.543-1.141)	0.207
***MT1G***							
Low	106	45	59	1		1	
High	106	46	57	0.865(0.601-1.246)	0.436	0.819(0.566-1.187)	0.292
***MT1F***							
Low	106	40	61	1		1	
High	106	51	55	0.824(0.572-1.187)	0.299	0.817(0.560-1.192)	0.294
***MT1E***							
Low	106	45	59	1		1	
High	106	46	57	0.896(0.622-1.289)	0.554	0.773(0.532-1.123)	0.177
***MT1HL1***							
Low	106	37	60	1		1	
High	106	51	56	0.836(0.581-1.204)	0.336	0.742(0.512-1.077)	0.116

**Notes**: §Adjusted for age, gender, cirrhosis, BCLC stage, serum AFP levels; RFS, recurrence-free survival; MRT, median recurrence time; HR, hazard ratio; CI, confidence interval. *PTTG1*, pituitary tumor-transforming 1; *TOP2A*, DNA topoisomerase II alpha, *PRC1*, protein regulator of cytokinesis 1, *MT2A*, metallothionein 2A, *MT1X*, metallothionein 1X, *MT1M*, metallothionein 1M, *MT1H*, metallothionein 1H, *MT1G*, metallothionein 1G, *MT1F*, metallothionein 1F, *MT1E*, metallothionein 1E, *MT1HL1*, metallothionein 1H like 1.

**Table 3:** Correlation between OS and 11 hub DEGs expression in HBV-related HCC patients of GSE14520 cohort

Variables	Patients (n=212)	MST (months)	No. of events	Crude HR (95% CI)	Crude P	Adjusted HR (95% CI)	Adjusted P§
**PTTG1**							
Low	106	NA	38	1		1	
High	106	NA	44	1.317(0.853-2.033)	0.215	1.390(0.893-2.166)	0.145
**PRC1**							
Low	106	NA	35	1		1	
High	106	57	47	1.731(1.116-2.685)	0.014	1.862(1.188-2.919)	0.007
**TOP2A**							
Low	106	NA	31	1		1	
High	106	53	51	2.107(1.347-3.296)	0.001	2.027(1.284-3.201)	0.002
**MT2A**							
Low	106	NA	42	1		1	
High	106	NA	40	0.943(0.611-1.454)	0.79	0.877(0.562-1.367)	0.562
**MT1X**							
Low	106	NA	43	1		1	
High	106	NA	39	0.858(0.556-1.324)	0.489	0.807(0.516-1.262)	0.347
**MT1M**							
Low	106	NA	44	1		1	
High	106	NA	38	0.824(0.534-1.273)	0.384	0.785(0.504-1.222)	0.284
**MT1H**							
Low	106	NA	45	1		1	
High	106	NA	37	0.769(0.498-1.189)	0.238	0.734(0.470-1.146)	0.174
**MT1G**							
Low	106	NA	42	1		1	
High	106	NA	40	0.885(0.574-1.365)	0.582	0.835(0.535-1.302)	0.426
**MT1F**							
Low	106	NA	45	1		1	
High	106	NA	37	0.782(0.506-1.209)	0.269	0.825(0.524-1.299)	0.406
**MT1E**							
Low	106	NA	43	1		1	
High	106	NA	39	0.863(0.560-1.332)	0.507	0.759(0.486-1.185)	0.225
**MT1HL1**							
Low	106	NA	45	1		1	
High	106	NA	37	0.756(0.489-1.168)	0.207	0.687(0.439-1.074)	0.1

**Notes**: §Adjusted for age, gender, cirrhosis, BCLC stage, serum AFP levels; OS, overall survival; MRT, median recurrence time; MST, median survival time; HR, hazard ratio; CI, confidence interval. *PTTG1*, pituitary tumor-transforming 1; *TOP2A*, DNA topoisomerase II alpha, *PRC1*, protein regulator of cytokinesis 1, *MT2A*, metallothionein 2A, *MT1X*, metallothionein 1X, *MT1M*, metallothionein 1M, *MT1H*, metallothionein 1H, *MT1G*, metallothionein 1G, *MT1F*, metallothionein 1F, *MT1E*, metallothionein 1E, *MT1HL1*, metallothionein 1H like 1.

**Table 4:** Survival analysis of risk score in HBV-related HCC patients of GSE14520 cohort

Variables	Patients (n=212)	MRT/MST(months)	No. of events	Crude HR (95% CI)	Crude *P*	Adjusted HR (95% CI)	Adjusted *P*§
**Risk score (RFS)**							
Low risk	106	54	52	1		1	
High risk	106	28	64	1.562(1.082-2.253)	0.017	1.525(1.045-2.224)	0.029
**Risk score (OS)**							
Low risk	106	NA	31	1		1	
High risk	106	52	51	2.140(1.367-3.349)	0.001	2.029(1.287-3.198)	0.002

**Notes**: §Adjusted for age, gender, cirrhosis, BCLC stage, serum AFP levels; RFS, recurrence-free survival; OS, overall survival; MRT, median recurrence time; MST, median survival time; HR, hazard ratio; CI, confidence interval. PRC1, protein regulator of cytokinesis 1; TOP2A, DNA topoisomerase II alpha.

**Table 5:** Correlation between OS and clinical features in HCC patients of TCGA cohort

Variables	Patients (n=370)	MST(days)	No. of events	HR (95% CI)	*P*
**Age ^£^**					
≤60	177	2532	55	1	
>60	190	1622	73	1.221(0.859-1.734)	0.265
**Gender**					
Female	121	1490	51	1	
Male	249	2486	79	0.817(0.573-1.164)	0.262
**Tumor stage^&^**					
I	171	2532	42	1	
II	85	1852	26	1.427(0.874-2.330)	0.155
III+IV	90	770	48	2.764(1.823-4.190)	<0.001

**Notes:** £ Information of age was unavailable in 3 patients; & Information of tumor stage was unavailable in 24 patients; MST, median survival time; HR, hazard ratio; CI, confidence interval; TCGA, The Cancer Genome Atlas.

**Table 6:** Survival analysis of *PRC1, TOP2A* and risk score in HCC patients of TCGA cohort

Variables	Patients (n=370)	MST(days)	No. of events	Crude HR (95% CI)	Crude *P*	Adjusted HR (95% CI)	Adjusted *P* ɠ
***PRC1* level**							
Low	185	2131	56	1		1	
High	185	1372	74	1.753(1.236-2.486)	0.002	1.817(1.235-2.673)	0.002
***TOP2A* level**							
Low	185	2116	57	1		1	
High	185	1397	73	1.701(1.200-2.410)	0.003	1.619(1.097-2.390)	0.015
**Risk score (OS)**							
Low risk	185	2131	57	1		1	
High risk	185	1397	73	1.754(1.237-2.486)	0.002	1.648(1.120-2.427)	0.011

**Notes**: ɠ Adjusted for age, gender, tumor stage; OS, overall survival; MST, median survival time; HR, hazard ratio; CI, confidence interval. PRC1, protein regulator of cytokinesis 1; TOP2A, DNA topoisomerase II alpha.
