# Supplementary material for: Comprehensive investigation of key biomarkers and pathways in hepatitis B virus-related hepatocellular carcinoma
Source: J Cancer. 2019 Sep 7;10(23):5689–704. doi: 10.7150/jca.31287 (PMC6843875; doi:10.7150/jca.31287)
Supplement: Supplementary file 1 — Supplementary figure and table legends, figures. [file jcav10p5689s1.pdf]

Supplementary material

Supplementary Figure legends

Figure S1. PRC1 and TOP2A expression in normal liver tissues base on GTEx database. PRC1 (A) and TOP2A (B) gene expression in multiple normal tissues.

Figure S2. PRC1 and TOP2A expression in HCC tumor and normal liver tissue base on The Human Protein Atlas database. Immunohistochemistry staining of PRC1 in normal liver (A) and HCC tumor tissue (B); Immunohistochemistry staining of TOP2A in normal liver (C) and HCC tumor tissue (D).

Figure S3. Comparison of TOP2A and PRC1 gene expression between tumor and adjacent normal tissue, and difference tumor stage in HCC patients. (A) Comparison between tumor and adjacent normal tissue in GSE14520 cohort; (B) Comparison between tumor and adjacent normal tissue in TCGA cohort; (C) Comparison among difference tumor stage in GSE14520 cohort; (D) Comparison difference tumor stage in TCGA cohort. The error bars represent mean and standard deviation.

Supplementary Tables

Table S1 GSEA result of HBV-related HCC tumor tissue using c5 reference gene sets.

Table S2 GSEA result of HBV-related HCC tumor tissue using c2 reference gene sets.

Table S3 Differentially expressed genes between HBV-related HCC tumor and adjacent normal tissue in GSE14520 cohort.

Table S4 GO term enrichment results of DEGs.

Table S5 KEGG enrichment results of DEGs.

Table S6 Results of weighted gene co-expression network.

Table S7 GSEA results of c5 reference gene sets for high PRC1 expression groups in HBV-related HCC patients of GSE14520 cohort.

Table S8 GSEA results of c2 reference gene sets for high PRC1 expression groups in HBV-related HCC patients of GSE14520 cohort.

Table S9 GSEA results of c5 reference gene sets for high TOP2A expression groups in HBV-related HCC patients of GSE14520 cohort.

Table S10 GSEA results of c2 reference gene sets for high TOP2A expression groups in HBV-related HCC patients of GSE14520 cohort.

Table S11 GSEA results of c5 reference gene sets for high PRC1 expression groups in HCC patients of TCGA cohort.

Table S12 GSEA results of c2 reference gene sets for high PRC1 expression groups in HCC patients of TCGA cohort.

Table S13 GSEA results of c5 reference gene sets for high TOP2A expression groups in HCC patients of TCGA cohort.

Table S14 GSEA results of c2 reference gene sets for high TOP2A expression groups in HCC patients of TCGA cohort.

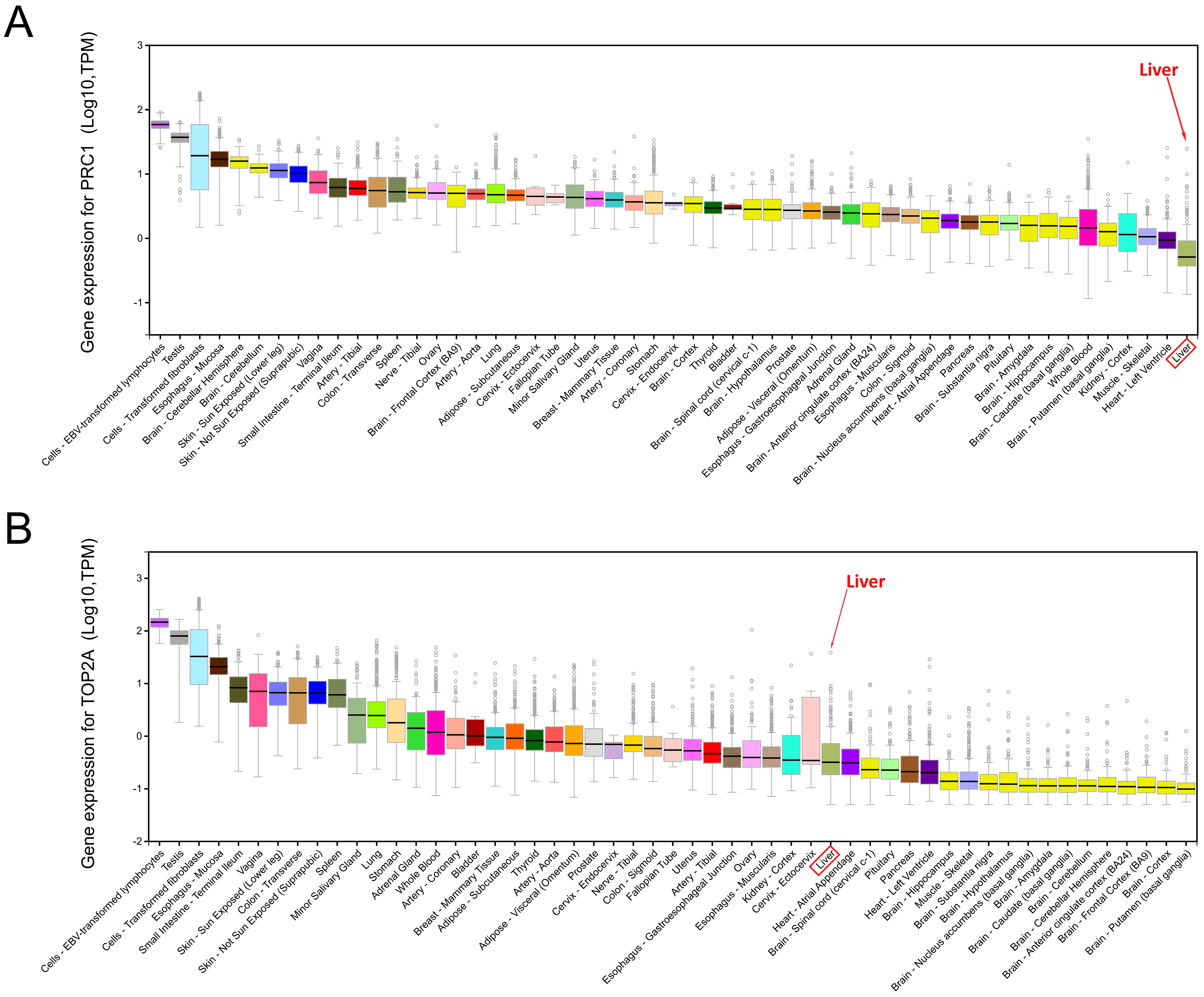

Figure S1. PRC1 and TOP2A expression in normal liver tissues base on GTEx database. PRC1 (A) and TOP2A (B) gene expression in multiple normal tissues.

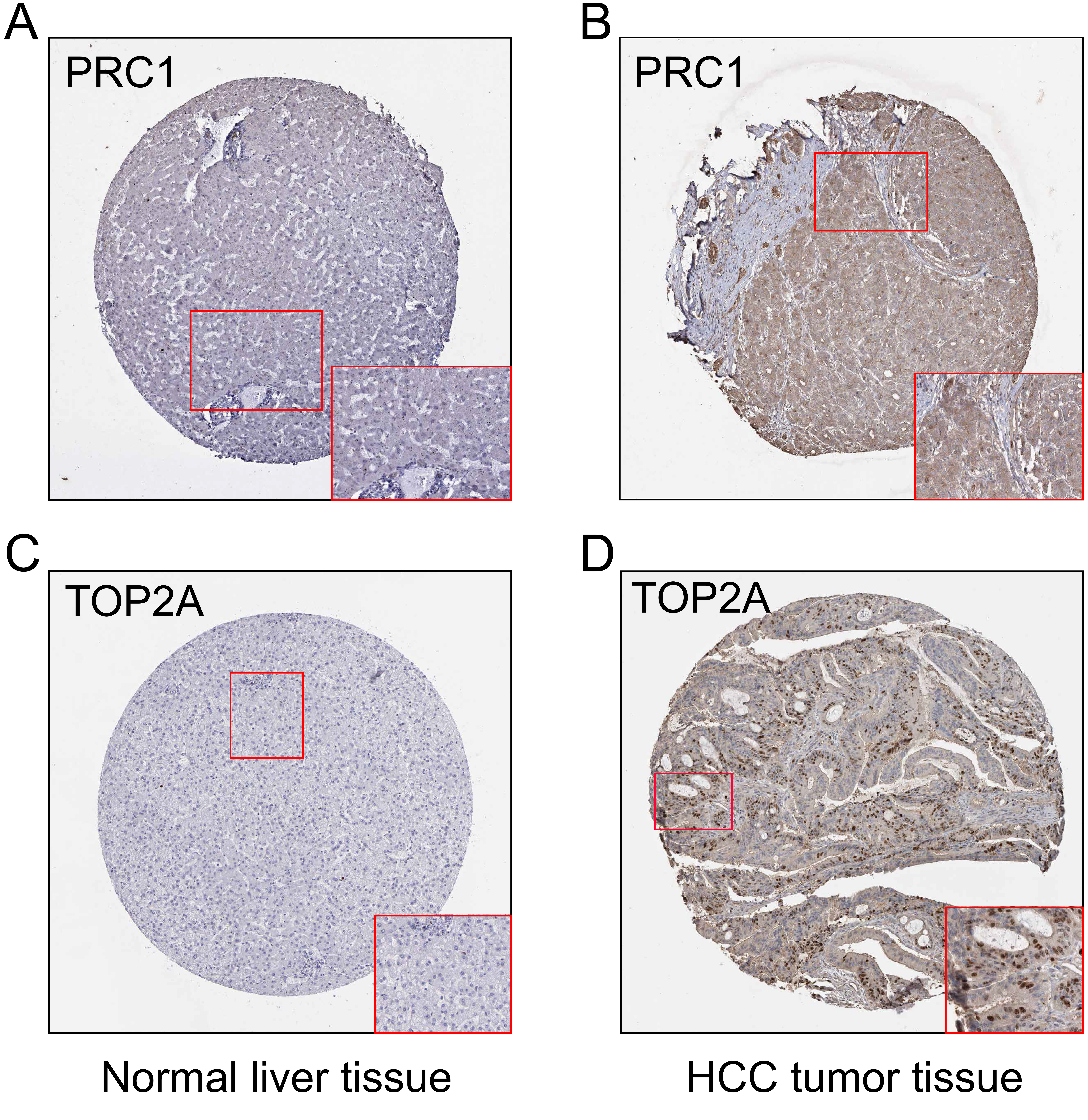

Figure S2. PRC1 and TOP2A expression in HCC tumor and normal liver tissue base onThe Human Protein Atlas database.
Immunohistochemistry staining of PRC1 in normal liver (A) and HCC tumor tissue (B); Immunohistochemistry staining of TOP2A in normal liver (C) and HCC tumor tissue (D).

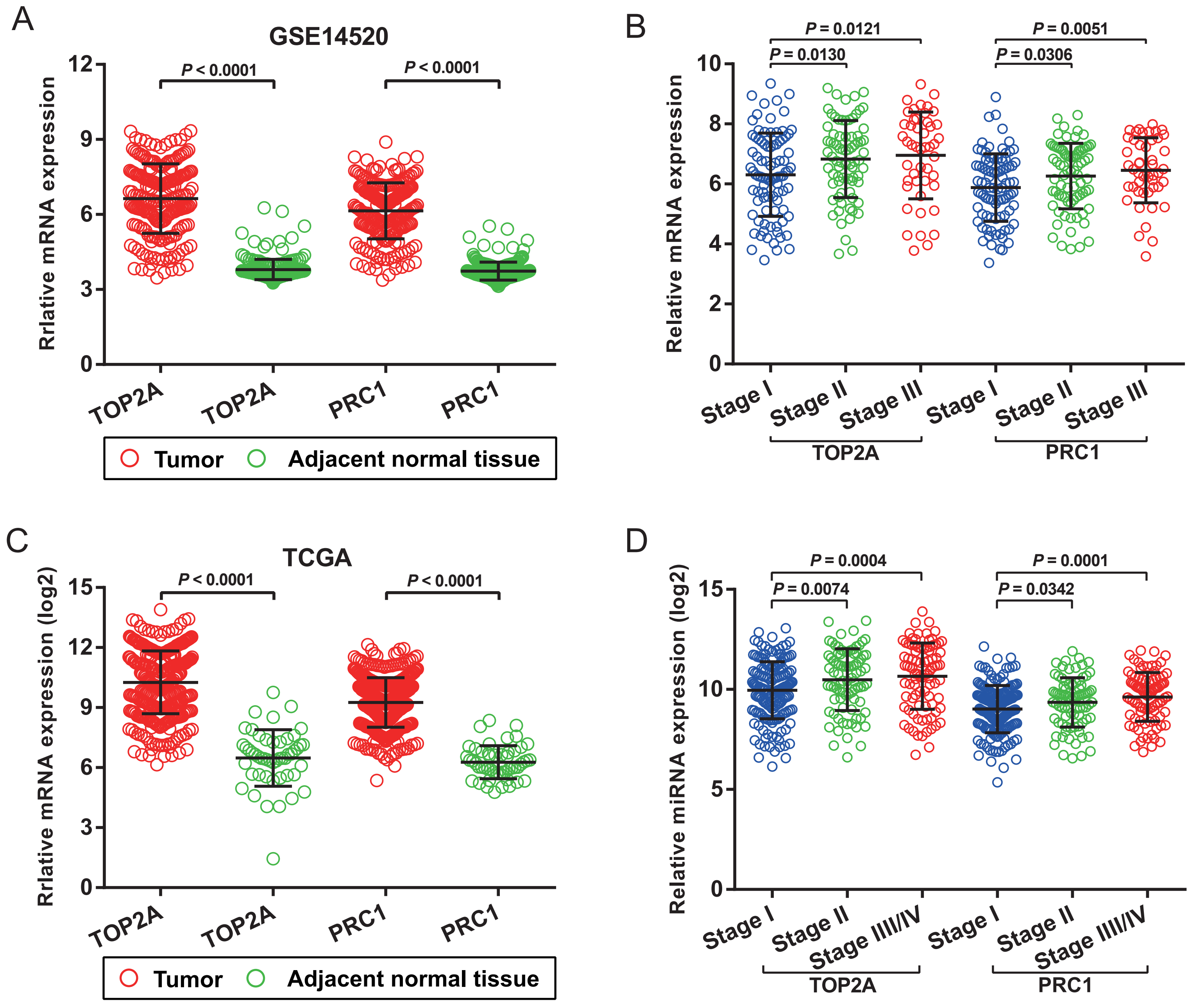

Figure S3. Comparison of TOP2A and PRC1 gene expression between tumor and adjacent normal tissue, and difference tumor stage in HCC patients.
(A) Comparison between tumor and adjacent normal tissue in GSE14520 cohort;
(B) Comparison between tumor and adjacent normal tissue in TCGA cohort;
(C) Comparison among difference tumor stage in GSE14520 cohort;
(D) Comparison difference tumor stage in TCGA cohort.
The error bars represent mean and standard deviation.
